# Supplementary material for: Dermatologic Simulation of Neglected Tropical Diseases for Medical Professionals
Source: MedEdPORTAL. 2016 Dec 31;12:10525. doi: 10.15766/mep_2374-8265.10525 (PMC6440398; doi:10.15766/mep_2374-8265.10525)
Supplement: Supplementary file 1 — A. Dengue Fever Simulation Case Template.docx B. Leishmaniasis Simulation Case Template.docx C. Lepromatous Leprosy Simulation Case Template.docx D. Yaws Simulation Case Template.docx E. Dermatological Door Sheets With Vital Signs.docx F. Standardized Patient Actor Scripts.docx G. Fact Sheets.docx H. Simulation Pictures.docx I. Postsimulation Survey.pdf [file mep-12-10525-s001.zip › I. Postsimulation Survey.pdf]

UCF Global Health Conference Simulations Survey

1. **Name (Optional)**

   ....

2. **Which best describes your profession?**
   Mark only one oval.

   - Medical Student
   - Nursing Student
   - Undergraduate Student
   - Pharmacy Student
   - Graduate Student
   - Professor/Physician
   - Other: ....

Dermatologic Simulation of Tropical Diseases

3. **Please indicate to what degree you agree or disagree with the following statements:**
   Mark only one oval per row.

	Strongly Disagree	Disagree	Neutral	Agree	Strongly Agree
This simulation improved my medical knowledge of Neglected Tropical Diseases					
This simulation improved my interpersonal communication Skills					
This simulation improved my critical thinking/diagnostic Skills					
This simulation experience was presented in a realistic environment					
This simulation was at an appropriate level					
The simulation facilitator(s) were enthusiastic, knowledgeable and helpful					
This simulation was useful to my profession					
This simulation increased my understanding of dermatological nomenclature					
Overall, this simulation was beneficial					

4. **If you have any general feedback about This Simulation/Workshop please indicate below:**

Powered by
Google Forms
