# Supplementary material for: Dermatologic Simulation of Neglected Tropical Diseases for Medical Professionals
Source: MedEdPORTAL. 2016 Dec 31;12:10525. doi: 10.15766/mep_2374-8265.10525 (PMC6440398; doi:10.15766/mep_2374-8265.10525)
Supplement: Supplementary file 1 — A. Dengue Fever Simulation Case Template.docx B. Leishmaniasis Simulation Case Template.docx C. Lepromatous Leprosy Simulation Case Template.docx D. Yaws Simulation Case Template.docx E. Dermatological Door Sheets With Vital Signs.docx F. Standardized Patient Actor Scripts.docx G. Fact Sheets.docx H. Simulation Pictures.docx I. Postsimulation Survey.pdf [file mep-12-10525-s001.zip › _Educational Summary Report_10525.pdf]

MedEdPORTAL®
The Journal of Teaching and Learning Resources

Original Publication

OPEN ACCESS

Dermatologic Simulation of Neglected Tropical Diseases for Medical Professionals

Michael Mankbadi, Laura Goyack*, Bryan Thiel, David Weinstein, MD, Judith Simms-Cendan, MD, Caridad Hernandez, MD
*Corresponding author: lgoyack@knights.ucf.edu

Citation: Mankbadi M, Goyack L, Thiel B, Weinstein D, Simms-Cendan J, Hernandez C. Dermatologic simulation of neglected tropical diseases for medical professionals. *MedEdPORTAL*. 2016;12:10525. https://doi.org/10.15766/mep_2374-8265.10525

Copyright: © 2016 Mankbadi et al. This is an open-access article distributed under the terms of the Creative Commons Attribution license.

Abstract

Introduction: While patients with neglected tropical diseases may present for care in the United States, they are encountered so infrequently that medical professionals may have little chance of being exposed to these illnesses during training. This simulation on dermatological presentations of neglected tropical diseases was used as a teaching tool for multifaceted topics including disease management, interdisciplinary collaboration, and sociocultural issues. To achieve this goal, we created four cases with patients demonstrating clinical and dermatological presentations. **Methods:** Through the use of a moulage kit, this simulation portrayed four common neglected tropical diseases that are rarely encountered in the United States: dengue fever, cutaneous leishmaniasis, lepromatous leprosy, and yaws. Following the clinical experience, a debrief session discussing sociocultural and dermatological factors of neglected tropical diseases occurred. **Results:** The feedback obtained regarding the simulation was exceptionally positive. The participants indicated that the simulation improved their medical knowledge of neglected tropical diseases, diagnostic abilities, and interprofessional communication skills. **Discussion:** This simulation can easily be adapted for use in conferences, health professional education, and patient advocacy, making it relevant for training in a wide variety of settings. Through the application of this simulation, greater progress can be made in the education of medical professionals on neglected tropical diseases and dermatology. Not only will the application of realistic workshops increase medical competency regarding these rarely encountered diseases, it will also provide opportunities to engage with these diseases, which can cultivate interest in the future pursuit of global health.

Keywords

Global Health, Neglected Tropical Diseases, Cutaneous Manifestations, Interprofessional Simulation

Appendices

A. Dengue Fever Simulation Case Template.docx
B. Leishmaniasis Simulation Case Template.docx
C. Lepromatous Leprosy Simulation Case Template .docx
D. Yaws Simulation Case Template.docx
E. Dermatological Door Sheets With Vital Signs .docx
F. Standardized Patient Actor Scripts.docx
G. Fact Sheets.docx
H. Simulation Pictures.docx
I. Postsimulation Survey.pdf

All appendices are peer reviewed as integral parts of the Original Publication.

Educational Objectives

By the end of this session, participants will be able to:

1. Describe the public health impact in the developing world of neglected tropical diseases and the reasons why clinicians in the United States should be able to recognize and correctly diagnose these conditions.
2. Use the information gathered from the medical history and physical examination of a standardized patient to correctly identify four common neglected tropical diseases.
3. Briefly summarize the epidemiology, symptoms, dermatologic findings, diagnostic workup, and treatment of eight common neglected tropical diseases.

Introduction

Nearly one billion of the world's population, often the impoverished, are infected with at least one neglected tropical disease (NTD).[1] The World Health Organization lists 15 NTDs, all of which are infectious, debilitating, and potentially lethal.[2] Moreover, nearly three billion people are at risk of contracting NTDs and do not have sufficient socioeconomic means to access treatment.[3,4] The lack of knowledge on this

topic in the medical community and society at large has led to increased complacency that will only perpetuate the presence of NTDs. There is a significant need to increase public and medical involvement in the eradication of NTDs.

However, little has been done on the integration of simulations and workshops in graduate education as a means to address this educational need. During the sixth annual Global Health Conference at the University of Central Florida's College of Medicine, seven simulation cases on NTDs were created and executed for the purpose of increasing awareness and knowledge of NTDs that may not be well understood because of their lack of prevalence in the medical community. Due to the wide variation of participant background knowledge, no prerequisite training or knowledge was required for the simulation. Our target audience included interprofessional graduate and undergraduate health students and staff from the fields of medicine, pharmacy, and nursing.

Although there are ample studies on the importance of dermatologic education, few resources are available for integrating NTDs. Previous team-based learning (TBL) exercises have taught common dermatologic characteristics with significant success. The Yale School of Medicine has implemented a Skin Signs of Systemic Disease TBL in its curriculum in order for learners to gain knowledge from written material and apply it to a clinical picture in real time at a clinical practice simulation.[5] Nevertheless, there is a lack of medical student and other health professional experience with NTDs and their dermatologic presentations.

Similarly, the Harvard Medical School offers a Clinical Topics in Global Health elective to students. This class combines topics with practical skills such as monitoring of active labor and basic tooth extraction.[6] All participants surveyed strongly agreed that "I would recommend this course to my classmates and colleagues."[6]

The need and desire for medical education on global health are strong in this country. Medical students' interest in global health has been increasing nationwide.[7] The goal of the Global Health Conference was to inform medical professionals of the importance of NTDs and encourage a lifelong appreciation for global health. This specific simulation focused on the dermatologic clinical presentation of these neglected diseases. By educating medical professionals on the dermatologic presentations, the importance of successful containment of NTDs can be brought before the public. Public awareness and media coverage may increase funding and community engagement to help foster pharmaceutical research and intervention strategies and increase the global effort necessary to eradicate NTDs.

Methods

The target audience for this resource is interprofessional graduate and undergraduate health students and staff from the fields of medicine, pharmacy, and nursing. Required personnel include four standardized patients, four makeup artists, one board-certified dermatologist, and three simulation directors.

Equipment required includes a Ben Nye professional makeup moulage kit, a hot-water heater needed for the liquid latex, a blow-dryer, cotton balls, paper clips, cups, Pond's cream lotion, laptop and projector, and clipboards with blank paper (at least one per participant).

Preparation

The three simulation directors designed the simulation to represent patients presenting with dermatologic manifestations of NTDs. We created four cases with patients demonstrating clinical presentations of dengue fever, cutaneous leishmaniasis, lepromatous leprosy, and yaws. The goal of the simulation was to have participants assess, diagnose, and improve medical knowledge regarding NTDs with dermatologic manifestations while working in an interdisciplinary team.

After designing the simulation, the first task was to prepare the four standardized patient actors for their roles. The standardized patient roles were played by four medical students. Two weeks prior to the simulation date, the patient scripts were finalized. They were emailed out to the standardized patient actors, ensuring adequate time for them to memorize and assimilate their roles. The scripts (Appendix F)

include details regarding the patients' onset of illness, symptoms, history, and ethnic information. The scripts were meant to be a guideline, and the patients were allowed to improvise if desired.

We recruited three medical students and one faculty member with varying backgrounds in moulage skills, ranging from little skill to an undergraduate degree in theater, to artistically portray the cutaneous lesions of dengue fever, cutaneous leishmaniasis, yaws, and lepromatous leprosy on the standardized patient actors.

One week before the simulation, we hosted a practice session for our moulage artists. We found instructions on how to use the moulage kit to represent yaws, cutaneous leishmaniasis, and dengue through various sources on the internet. Based on the practice session, we determined that 2 hours would be necessary for setup on the day of the simulation. See Appendix H to view photographs of the moulage disease replications. Moreover, for future replications of this simulation, it would be highly beneficial to have another blinded dermatologist view the moulaged patients and confirm that the dermatological presentations are accurate.

Logistics

The simulation was held in the University of Central Florida College of Medicine Clinical Skills Simulation Center to represent realistic patient encounters. This allowed the participants to have access to clinic resources such as ophthalmoscopes, reflex hammers, and blood pressure monitors as needed to perform a physical exam on the standardized patients. However, these clinic resources are not essential for the simulation and can be easily adapted for future replications.

The simulation lasted a total of 50 minutes and was broken down into the following time line. Each group consisted of a maximum of four students, and each group visited a total of four rooms representing four different diseases:

- Three minutes: introduction by conference host to explain the simulation and divide the participants into groups.
- Twenty-four minutes: 6 minutes per room times four rooms.
- Three minutes: walk to student group learning room, located in a different area of the building.
- Five minutes: debrief portion to allow the students to diagnose the four diseases.
- Fifteen minutes: debrief with a PowerPoint by the dermatologist.
- Postsimulation survey received by email (Appendix I).

These time limits were determined with the aim of allowing maximum time for interviewing patients and discussion within the allotted total 50 minutes. The simulation templates for each disease portrayed by the standardized patient actors can be found in Appendices A-D.

Upon arriving at the patient room, participants found a sheet with vital signs and physical exam findings (Appendix E) posted on the door. This allowed the participants to receive expedited pieces of information, necessary because of the limited window of time available to assess each of the standardized patients. Each participant received a clipboard with blank sheets of paper to take notes. The participants interviewed, examined, and assessed the standardized patients. After evaluation of each patient, participants were handed a fact sheet on either epidemiology, photographs of dermatologic presentation, treatment, common symptoms, or diagnostic workup (Appendix G). After groups had assessed all of the standardized patient actors and received all five fact sheets, there was a 5-minute session to work together to discuss differential diagnoses for each case. The participants used the fact sheets to synthesize information received from the standardized patients and come up with a diagnosis. Afterwards, these results were checked and discussed with a board-certified dermatologist regarding the correct disease association, with an emphasis on the dermatologic presentation.

Assessment and Debriefing

After the 30-minute simulation came a 20-minute postsession debrief, which we believe was beneficial in encouraging solidification of the material. The first 5 minutes of this debrief were dedicated to allowing the students to work together with their notes and fact sheets to diagnose the four dermatological

diseases they had witnessed. Then, a 15-minute small-group discussion with a PowerPoint debrief was led by the dermatologist. The dermatologist discussed the findings and purported diagnoses for each standardized patient to assess for accuracy and thought process in assessment. The PowerPoint emphasized important topics for the students to take from the simulation. The dermatologist assessed each of the groups and hosted a roundtable discussion to further elaborate on techniques and methods to accurately diagnose these tropical diseases. A general question-and-answer session was incorporated throughout the presentation. A postconference survey was emailed to the participants after the conference to determine whether they had an increased knowledge and skill set with which to assess the diseases and other education objectives.

Results

Thirty-seven conference participants attended this simulation. A postsimulation survey was sent out to all of the individuals who attended the workshop portion of the conference (Appendix I). The survey entailed rating the simulation on a 5-point Likert scale (1 = *strongly disagree,* 5 = *strongly agree*) with a list of statements. Of those who completed the survey, 38.5% were medical students, 46.0% were undergraduate students, 7.7% were medical faculty, and 7.7% listed their profession as other.

The average rating for whether the simulation improved the participants' medical knowledge of NTDs was 4.38. Likewise, participants rated the statement that the simulation improved their interpersonal communication skills as 4.17. In regard to the participants' improvement in critical thinking and diagnostic skills, the average rating was 4.50. Participants agreed that the simulation was useful to their profession and that it increased their knowledge of dermatologic nomenclature, with respective ratings of 4.33 and 4.17. The survey also indicated that the majority of participants found the simulation presented a realistic environment at an appropriate level. Overall, many participants strongly agreed that the simulation was beneficial, with an average rating of 4.50 out of 5.

Written and oral qualitative feedback was also positive. One participant stated, "I found the clinical simulation extremely valuable to provide information that I would not normally encounter." Another said, "I really enjoyed trying to diagnose the patients!"

We had a pilot test run before the simulation date. From this session, we determined which dermatologic conditions could be quickly and effectively moulaged. We also discovered the amount of time needed to prepare the moulage was significantly longer than we had expected, and therefore, we planned accordingly on the day of the simulation. It was beneficial to have a practice moulage session and to designate what every moulage artist would be doing the day of the simulation. The faculty facilitator gave a practice debrief session in which we tested all of the technologic equipment and debrief dynamics. We found it was best to organize the debrief session into the same groups that the participants had gone through in the simulation.

Discussion

Overall, this is an effective and realistic clinical application of NTDs that can be implemented in any program. There is much flexibility in the setup, and it can be adapted for nontropical dermatologic diseases. This simulation was specifically created for educational purposes at the 2016 Global Health Conference at the University of Central Florida College of Medicine. While the diseases simulated in our workshop may not often be seen in the United States, they nevertheless have a tremendous global impact. It is important to raise awareness of NTDs, especially now that globalization of disease is becoming more and more prevalent (e.g., Ebola virus, Zika virus).

We can start educating our medical professionals to be more competent in these rare diseases through realistic workshops, which will prepare them for the future. Furthermore, providing opportunities to engage with these diseases can cultivate interest in the future pursuit of global health. To accomplish these goals, simulations should be highly engaging in order to make an impact on the participants.

The preparation of this simulation on the day of administration is dependent only on the length of time necessary to apply the moulage kit to the standardized patients. The remaining tasks, including the creation of door sheets, fact sheets, and actor scripts, should be completed ahead of time and these items provided to the standardized patients in advance so they will have familiarity with what to expect from participants as regards questions and physical exam maneuvers. The greatest amount of attention should be directed towards the creation of the fact sheets, as most participants will not have seen or know much about each of the tropical diseases. The fact sheets located in Appendix G are provided to expedite future replications of this simulation and to reduce the time necessary for preparation. We created the fact sheets to provide participants with enough information to determine the disease when they were synthesized and cross-referenced with the actor scripts the standardized patients utilized. The resources from which we obtained most of the information for the fact sheets can be found on UpToDate, Medscape, and the Centers for Disease Control and Prevention Web sites.

For our workshop, the biggest challenge was the time constraints, due to the rigidity of the Global Health Conference schedule. We created this simulation to run for 50 minutes, but we would encourage future simulations to extend the total time to provide an even more meaningful and engaged experience with these tropical diseases. Similarly, the time can be expanded to include more than four cutaneous diseases.

Another limitation to this simulation may be that individuals who have not had experience in taking a history and physical could feel uneasy given the nature of the simulation. We did our best to limit each group to three to four participants, as larger groups might hinder individual participation. Moreover, there was a wide range of background knowledge in some of the groups, with more-experienced individuals taking a more-dominant role. This can possibly be overcome by pairing individuals of similar medical backgrounds together.

We found that with the moulage kit, the diseases selected worked well. We would suggest practicing with the makeup artists ahead of time to ensure that the diseases can be realistically demonstrated on the standardized patients. While this adds additional preparation work, we found that it greatly increased the efficiency in setup on the day of the simulation. For future replications of this simulation, it would be highly beneficial to have another blinded dermatologist view the moulaged patients and confirm that the dermatological presentations are accurate. This would improve internal reliability for future recreations.

As seen in the Appendices, a significant amount of time is required to create the fact sheets, but this was deemed appropriate and necessary due to the varied levels of participants' previous knowledge of these NTDs. Having the fact sheets also allowed participants to take home physical pieces of information that they could use in the future. The total time spent on preparation, including creation of the fact sheets, standardized patient recruitment, faculty recruitment, and overall planning of the simulation, was roughly 100 hours. Our goal here is to provide enough insight so that this simulation can be readily assembled and executed in approximately 15 hours.

Evaluation was assessed through the roundtable discussion during debriefing with a board-certified dermatologist who provided feedback to participants to clarify major points about each of the cutaneous diseases. Alternatively, pre- and postsurveys could be utilized in the future to assess whether the simulation provides increased knowledge of the clinical presentation and diagnostic workup of NTD. A postsimulation quiz may be employed, but due to the time constraints on our program, we chose to forgo this route. However, we believe implementation of quizzes will encourage participant engagement and knowledge retention. The participants who attended this program appreciated and enjoyed the realistic and immersive experience of being a medical care provider. Many were not expecting to see such realistic portrayals of NTDs, and as a result, this empowered them to do their utmost to provide the best care possible. Overall, we feel very strongly that this simulation successfully reached the educational objectives of interest.

Michael Mankbadi: Medical Student, University of Central Florida College of Medicine

Laura Goyack: Medical Student, University of Central Florida College of Medicine

Bryan Thiel: Medical Student, University of Central Florida College of Medicine

David Weinstein, MD: Assistant Professor of Dermatology, University of Central Florida College of Medicine

Judith Simms-Cendan, MD: Associate Professor of Obstetrics and Gynecology, University of Central Florida College of Medicine; Director of International Experiences, University of Central Florida College of Medicine

Caridad Hernandez, MD: Associate Professor of Internal Medicine, University of Central Florida College of Medicine

Disclosures
None to report.

Funding/Support
None to report.

Ethical Approval
Reported as not applicable.

References

1. Neglected tropical diseases. Centers for Disease Control and Prevention Web site. http://www.cdc.gov/globalhealth/ntd/. Updated June 7, 2016. Accessed June 19, 2016.
2. Hotez PJ, Fenwick A, Savioli L, Molyneux DH. Rescuing the bottom billion through control of neglected tropical diseases. *Lancet.* 2009;373(9674):1570-1575. https://doi.org/10.1016/S0140-6736(09)60233-6
3. Neglected tropical diseases. World Health Organization Web site. http://www.who.int/neglected_diseases/en/. Accessed May 10, 2016.
4. Why NTDs? END7 Web site. http://www.end7.org/why-ntds. Accessed May 10, 2016.
5. Chapman R, Sabina R, Thomas D. Skin signs of systemic disease TBL. *MedEdPORTAL Publications.* 2015;11:10184. http://doi.org/10.15766/mep_2374-8265.10184
6. Lee P, Johnson A, Rajashekara S, et al. Clinical topics in global health: a practical introduction for pre-clinical medical students. *MedEdPORTAL Publications*. 2013;9:9471. http://doi.org/10.15766/mep_2374-8265.9471
7. Panosian C, Coates TJ. The new medical “missionaries”—grooming the next generation of global health workers. *N Engl J Med.* 2006;354(17):1771-1773. https://doi.org/10.1056/NEJMp068035

Received: August 15, 2016 | **Accepted:** December 15, 2016 | **Published:** December 31, 2016
