# Supplementary material for: Dermatologic Simulation of Neglected Tropical Diseases for Medical Professionals
Source: MedEdPORTAL. 2016 Dec 31;12:10525. doi: 10.15766/mep_2374-8265.10525 (PMC6440398; doi:10.15766/mep_2374-8265.10525)
Supplement: Supplementary file 1 — A. Dengue Fever Simulation Case Template.docx B. Leishmaniasis Simulation Case Template.docx C. Lepromatous Leprosy Simulation Case Template.docx D. Yaws Simulation Case Template.docx E. Dermatological Door Sheets With Vital Signs.docx F. Standardized Patient Actor Scripts.docx G. Fact Sheets.docx H. Simulation Pictures.docx I. Postsimulation Survey.pdf [file mep-12-10525-s001.zip › A. Dengue Fever Simulation Case Template.docx]

| **Appendix A: MedEdPORTAL Simulation Case Template**  **SIMULATION CASE TITLE:** Dengue Fever Dermatology Simulation  **AUTHORS:** Michael Mankbadi, BS, Laura Goyack, BS, Bryan Thiel, BS,  David Weinstein, MD, Judith Simms-Cendan, MD, Caridad Hernandez, MD | |
| --- | --- |
| **PATIENT NAME: Sagar Siva**  **PATIENT AGE: 23-year-old**  **CHIEF COMPLAINT: Rash and fever** | |
| **Brief narrative description of case** | The patient is a 23-year-old male who has had a petechial rash for four days. The patient also has noticed fever, joint pain, and headache. The objective for this scenario is part of a set of four simulations in which participants will have a greater understanding of the dermatologic aspects of neglected tropical diseases |
| **Primary Learning Objectives** | The learning objectives for this simulation are that participants will be able to better:   - Describe, assess, and diagnose patients presenting with common neglected tropical diseases with dermatologic manifestations - Learn the nomenclature used to describe dermatologic manifestations - Improve clinical skills and communication through interactions with standardized patients - Understand the global health significance of these tropical diseases - Work in an inter-professional group, in a way that respects patient autonomy while limiting medical jargon |
| **Critical Actions** | 1. Participants will work together as an inter-professional team to take a thorough history of a patient presenting with Dengue Fever. 2. Participants will interact professionally with the patient. 3. Participants will use their notes regarding this patient to correctly diagnose Dengue Fever in the debrief session. |
| **Learner Preparation** | No prior information needed. |

| **Initial Presentation** | | | |
| --- | --- | --- | --- |
| **Initial vital signs** | Heart Rate: 80 bpm  Respiratory Rate: 10 breaths per minute  Temperature: 103.0^o^ F  Blood Pressure: 118/76 | | |
| **Overall Appearance** | The patient is alert, in no acute distress, and sitting on the exam table. A noticeable petechial rash is present on his back and right arm. The room is stocked with gowns, drapes, gloves, and other physical exam components. | | |
| **Actors and roles in the room at case start** | The actor can be any gender, age and ethnicity and they will have the rash as described in the overall appearance section above. Their role is to act as a patient with Dengue Fever. May be played by health professional student or professional standardized patient. | | |
| **HPI** | **HPI:**  Volunteered by patient-  The patient has had a “spotty” rash for four days that originally started on his back and then has recently appeared on his right arm.  Upon elicitation by participants-  -Patient also notices fever, joint pain, and headache.  **-**He recently had vacationed in Panama and came back last week.  -He is a nature enthusiast and was in parts of the jungle  **ROS:**  - General: fever  - HEENT: Headache, pain behind eyes.  - Musculoskeletal: painful joints, muscle pain.  **Social History:**  - Exercises regularly  - Balanced Diet  - Smokes 1 pack/week  - EtOH: 8 drinks/week (especially on weekends)  - Occupation: Graduate student studying botany  - Living arrangement: With roommates at a dorm.  - Not sexually active | | |
| **Past Medical/Surgical History** | **Medications** | **Allergies** | **Family History** |
| - No past illnesses, surgeries, or hospitalizations - No immunizations or preventative screening done | None | None | - Parents and two younger brothers are living, but in small village in India. - No known diseases in the family |
| **Physical Examination:** | | | |
| **General** | Well-developed, well-nourished male. No acute distress. | | |
| **HEENT** | Bleeding gums | | |
| **Neck** |  | | |
| **Lungs** |  | | |
| **Cardiovascular** |  | | |
| **Abdomen** |  | | |
| **Neurological** |  | | |
| **Skin** | Numerous scattered petechiae on right arm and back. | | |
| **GU** |  | | |
| **Psychiatric** |  | | |

| **Instructor Notes - Changes and Case Branch Points**  Due to the nature of this simulation this section is not necessary. | | |
| --- | --- | --- |
| **Intervention / Time point** | **Change in Case** | **Additional Information** |
| *6 minutes into the interview* | *Termination of the interview* |  |

**Ideal Scenario Flow**

The simulation participants have a minute to read the information posted outside of the patient encounter room that displays vitals and physical exam findings that cannot be demonstrated on the patient. The simulation participants enter the room to a find a patient in no acute distress, but with a petechial rash is found on his back and right arm. They begin to obtain a history from the patient learning about his social history and especially the patients recent travels to Panama. The participants take notes regarding the dermatologic presentation of the patient, history, epidemiology, and symptoms.

At the conclusion of the six minutes per room, the patient will break character and present the learners with a fact sheet containing useful information for diagnosing dermatologic neglected tropical diseases. The simulation participants are then directed to the next patient room, where the process is repeated with a different patient encounter. The participants gather a total of five fact sheets from the four patient rooms. Using the fact sheets, they will be able to identify which disease this patient had by compiling their notes as a group.

**Anticipated Management Mistakes**

Due to the quick paced nature of the encounter, we anticipated that the standardized patient might forget to present the fact sheet to the exiting group. This did not happen, but it should be stressed to the standardized patients to not forget.

A mistake encountered during the simulation was that occasionally one individual in the group would be take a dominant role, limiting other individual interaction. This would likely not be as significant in pre-established groups or groups of individuals with the same background knowledge.
