# Supplementary material for: Dermatologic Simulation of Neglected Tropical Diseases for Medical Professionals
Source: MedEdPORTAL. 2016 Dec 31;12:10525. doi: 10.15766/mep_2374-8265.10525 (PMC6440398; doi:10.15766/mep_2374-8265.10525)
Supplement: Supplementary file 1 — A. Dengue Fever Simulation Case Template.docx B. Leishmaniasis Simulation Case Template.docx C. Lepromatous Leprosy Simulation Case Template.docx D. Yaws Simulation Case Template.docx E. Dermatological Door Sheets With Vital Signs.docx F. Standardized Patient Actor Scripts.docx G. Fact Sheets.docx H. Simulation Pictures.docx I. Postsimulation Survey.pdf [file mep-12-10525-s001.zip › B. Leishmaniasis Simulation Case Template.docx]

| **Appendix B: MedEdPORTAL Simulation Case Template**  **SIMULATION CASE TITLE:** Leishmaniasis Dermatology Simulation  **AUTHORS:** Michael Mankbadi, BS, Laura Goyack, BS, Bryan Thiel, BS,  David Weinstein, MD, Judith Simms-Cendan, MD, Caridad Hernandez, MD | |
| --- | --- |
| **PATIENT NAME:  Joanne Jones**  **PATIENT AGE: 26-year-old**  **CHIEF COMPLAINT:  Ulcer on right arm** | |
|  | |
| **Brief narrative description of case**  *Include the presenting patient chief complaint* | The patient is a 26-year-old female that presents with a chief complaint of an ulcer on right arm. The objective for this scenario is part of a set of four simulations in which participants will have a greater understanding of the dermatologic aspects of neglected tropical diseases. |
| **Primary Learning Objectives** | The learning objectives for this simulation are that patients will be able to better:   - Describe, assess, and diagnose patients presenting with common neglected tropical diseases with dermatologic manifestations - Learn the nomenclature used to describe dermatologic manifestations - Improve clinical skills and communication through interactions with standardized patients - Understand the global health significance of these tropical diseases - Work in an inter-professional group, in a way that respects patient autonomy while limiting medical jargon |
| **Critical Actions** | 1. Participants will work together as an inter-professional team to take a   thorough history of a patient presenting with Leishmaniasis.   1. Participants will interact professionally with the patient. 2. Participants will use their notes regarding this patient to correctly diagnose leishmaniasis in the debrief session. |
| **Learner Preparation** | No prior information needed. |

| **Initial Presentation** | | | |
| --- | --- | --- | --- |
| **Initial vital signs** | Heart Rate: 68 bpm  Respiratory Rate: 12 breaths per minute  Temperature: 98.6^o^ F  Blood Pressure: 122/78 | | |
| **Overall Appearance** | The patient is alert, in no acute distress and sitting on the exam table. The room is stocked with gowns, drapes, gloves, and other physical exam components. The patient has a noticeable ulcer on their arm. | | |
| **Actors and roles in the room at case start** | One actor playing the patient. May be played by health professional student or professional standardized patient. | | |
| **HPI** | **HPI:** Volunteered by patient-  First noticed small erythematous bite marks on arms and ankles. Thought they were simply bug bites so she didn’t do anything about it.  Upon elicitation by participants-  Bites became enlarged and nodular. She noticed that the surrounding skin became tough and the color changed to a white/yellow. The bites opened up and now have pus and drainage. She was on a medical mission trip to Bolivia three weeks ago. | | |
| **Past Medical/Surgical History** | **Medications** | **Allergies** | **Family History** |
| No past illnesses, surgeries, or hospitalizations | Advil as needed. Tried herbal medicines in Bolivia. | No known allergies | No relevant family history |
| **Physical Examination-** | | | |
| **General** | Well-developed, well-nourished. No acute distress. | | |
| **HEENT** |  | | |
| **Neck** |  | | |
| **Lungs** |  | | |
| **Cardiovascular** |  | | |
| **Abdomen** | Hepatomegaly. | | |
| **Neurological** |  | | |
| **Skin** | Ulcer with violaceous border on right arm. | | |
| **GU** |  | | |
| **Psychiatric** |  | | |

| **Instructor Notes - Changes and Case Branch Points**  Due to the nature of this simulation this section is not necessary. | | |
| --- | --- | --- |
| **Intervention / Time point** | **Change in Case** | **Additional Information** |
| *6 minutes into the interview* | *Termination of the interview* |  |

**Ideal Scenario Flow**

The simulation participants enter the room to a find a patient in no immediate distress, but with a significant ulcer upon examination. They begin to obtain a history from the patient. learning about her social history and note that the patient has recently traveled to Bolivia. The participants take notes regarding the dermatologic presentation of the patient, history, epidemiology, and symptoms. At the conclusion of the six minutes per room, the patient will break character and present the participants with a fact sheet containing useful information for diagnosing dermatologic neglected tropical diseases.

The simulation participants are then directed to the next patient room, where the process is repeated with a different patient encounter. The participants gather a total of five fact sheets from the four patient rooms. Using the fact sheets, they will be able to identify which disease this patient had by compiling their notes as a group.

**Anticipated Management Mistakes**

Due to the quick paced nature of the encounter, we anticipated that the standardized patient might forget to present the fact sheet to the exiting group. This did not happen, but it should be stressed to the standardized patients to not forget.

A mistake encountered during the simulation was that occasionally one individual in the group would be take a dominant role, limiting other individual interaction. This would likely not be as significant in pre-established groups or groups of individuals with the same background knowledge.
