# Supplementary material for: Dermatologic Simulation of Neglected Tropical Diseases for Medical Professionals
Source: MedEdPORTAL. 2016 Dec 31;12:10525. doi: 10.15766/mep_2374-8265.10525 (PMC6440398; doi:10.15766/mep_2374-8265.10525)
Supplement: Supplementary file 1 — A. Dengue Fever Simulation Case Template.docx B. Leishmaniasis Simulation Case Template.docx C. Lepromatous Leprosy Simulation Case Template.docx D. Yaws Simulation Case Template.docx E. Dermatological Door Sheets With Vital Signs.docx F. Standardized Patient Actor Scripts.docx G. Fact Sheets.docx H. Simulation Pictures.docx I. Postsimulation Survey.pdf [file mep-12-10525-s001.zip › C. Lepromatous Leprosy Simulation Case Template.docx]

| **Appendix C: MedEdPORTAL Simulation Case Template**  **SIMULATION CASE TITLE:** Lepromatous Leprosy Dermatology Simulation  **AUTHORS:** Michael Mankbadi, BS, Laura Goyack, BS, Bryan Thiel, BS,  David Weinstein, MD, Judith Simms-Cendan, MD, Caridad Hernandez, MD | |
| --- | --- |
| **PATIENT NAME: Javier Vasconsellos**  **PATIENT AGE: 34-year-old**  **CHIEF COMPLAINT: Painless facial lesions on face and trunk that are associated with numbness** | |
| **Brief narrative description of case** | The patient is a 34-year-old male that presents with a raised, waxy, tumor-like lesion on the face that is associated with numbness to reflect lepromatous leprosy dermatologic changes. The objective for this scenario is part of a set of four simulations in which participants will have a greater understanding of the dermatologic aspects of neglected tropical diseases. |
| **Primary Learning Objectives** | The learning objectives for this simulation are that participants will be able to better:   - Describe, assess, and diagnose patients presenting with lepromatous leprosy. - Learn the nomenclature used to describe dermatologic manifestations - Improve clinical skills and communication through interactions with standardized patients - Understand the global health significance of these tropical diseases - Work in an inter-professional group, in a way that respects patient autonomy while limiting medical jargon. |
| **Critical Actions** | 1. Participants will work together as an inter-professional team to take a thorough history of a patient presenting with lepromatous leprosy. 2. Participants will interact professionally with the patient. 3. Participants will use their notes regarding this patient to correctly diagnose lepromatous leprosy in the debrief session. |
| **Learner Preparation** | No prior information is needed. |

| **Initial Presentation** | | | |
| --- | --- | --- | --- |
| **Initial vital signs** | Heart Rate: 66 bpm  Respiratory Rate: 10 breaths per minute  Temperature: 98.6^o^ F  Blood Pressure: 126/82 | | |
| **Overall Appearance** | The patient is sitting in the room, with large, waxy, tumor-like raised lesions on the face. The hair is disheveled and the patient has difficulty closing the eyes due to the size and location of the lesion. At the collar of the shirt, there are similar thin plaques on the chest. The patient’s feet have few cuts and bruising. | | |
| **Actors and roles in the room at case start** | The actor can be any gender, age and ethnicity and they will have the lesions as described in the overall appearance section above. Their role is to act as a patient with lepromatous leprosy. May be played by health professional student or professional standardized patient. | | |
| **HPI** | **HPI:**  Volunteered by patient-  -The patient noticed these changes beginning approximately ten years ago  -Started noticing more skin patches on trunk  -Lesions spread to face that he is seeking help  Upon elicitation by participants-  -Progressive loss of sensation in patches  **ROS:**  -Swelling in hands and slight joint pain in fingers  -Peripheral neuropathy in hands/feet  -Ulnar nerve problems (failure in movement of 4^th^/5^th^ digit)  -Trouble closing eyes  -Hoarseness of voice  -Nose deformation  **Social History:**  **-** Patient was born and raised in Brazil and moved to the United  States 2 weeks ago.  - Divorced  - Exercises regularly  - Balanced Diet  - Smokes 1pack/week  - EtOH: Few beers on the weekend.  - Occupation: Unemployed  - Education: Graduated from high school  - Living arrangement: Alone in small 1-bedroom apartment.  - Not sexually active | | |
| **Past Medical/Surgical History** | **Medications** | **Allergies** | **Family History** |
| - No past illnesses, surgeries, hospitalizations, - No immunizations and preventative screenings | Advil and herbal medicines PRN | No known allergies | There are no known chronic illnesses in the family. The parents and two siblings are all still living in a small village in Brazil. |
| **Physical Examination:** | | | |
| **General** | Fatigue, no acute distress. | | |
| **HEENT** | Destruction of nasal cartilage. | | |
| **Neck** |  | | |
| **Lungs** |  | | |
| **Cardiovascular** | Bilateral hand edema. | | |
| **Abdomen** |  | | |
| **Neurological** | Ulnar nerve weakness, Peripheral neuropathy in hands/feet. | | |
| **Skin** | Thick skin-colored tumors and plaques on the forehead and brow. Thinner skin-colored plaques on chest. | | |
| **GU** |  | | |
| **Psychiatric** |  | | |

| **Instructor Notes - Changes and Case Branch Points**  Due to the nature of this simulation this section is not necessary. | | |
| --- | --- | --- |
| **Intervention / Time point** | **Change in Case** | **Additional Information** |
| *6 minutes into the case* | *Terminate the encounter.* |  |

**Ideal Scenario Flow**

The simulation participants have a minute to read the information posted outside of the patient encounter room that displays vitals and physical exam findings that cannot be demonstrated on the patient. The simulation participants enter the room to a find a patient in no immediate distress, but with facial lesions upon examination. They enter the room and spend six minutes interviewing the patient for the history of present illness, past medical history, family history and social history.

At the conclusion of the encounter, the patient presents a fact sheet that contains useful information for diagnosing dermatologic neglected tropical diseases. The simulation participants are then directed to the next patient room, where the process is repeated with a different patient encounter. The participants gather a total of five fact sheets from the four patient rooms. Using the fact sheets, they will be able to identify which disease this patient had by compiling their notes as a group.

**Anticipated Management Mistakes**

Due to the quick paced nature of the encounter, we anticipated that the standardized patient might forget to present the fact sheet to the exiting group. This did not happen, but it should be stressed to the standardized patients to not forget.

A mistake encountered during the simulation was that occasionally one individual in the group would be take a dominant role, limiting other individual interaction. This would likely not be as significant in pre-established groups or groups of individuals with the same background knowledge.
