# Supplementary material for: Dermatologic Simulation of Neglected Tropical Diseases for Medical Professionals
Source: MedEdPORTAL. 2016 Dec 31;12:10525. doi: 10.15766/mep_2374-8265.10525 (PMC6440398; doi:10.15766/mep_2374-8265.10525)
Supplement: Supplementary file 1 — A. Dengue Fever Simulation Case Template.docx B. Leishmaniasis Simulation Case Template.docx C. Lepromatous Leprosy Simulation Case Template.docx D. Yaws Simulation Case Template.docx E. Dermatological Door Sheets With Vital Signs.docx F. Standardized Patient Actor Scripts.docx G. Fact Sheets.docx H. Simulation Pictures.docx I. Postsimulation Survey.pdf [file mep-12-10525-s001.zip › D. Yaws Simulation Case Template.docx]

| **Appendix D: MedEdPORTAL Simulation Case Template**  **SIMULATION CASE TITLE:** Yaws Dermatology Simulation  **AUTHORS:** Michael Mankbadi, BS, Laura Goyack, BS, Bryan Thiel, BS,  David Weinstein, MD, Judith Simms-Cendan, MD, Caridad Hernandez, MD | |
| --- | --- |
| **PATIENT NAME: Aban Imari**  **PATIENT AGE: 15-year-old**  **CHIEF COMPLAINT: Yellow crusty lesion on right arm** | |
| **Brief narrative description of case** | The patient is a 15-year-old female presenting with a yellow crusting lesion on the right arm and muscle pains. The goal of this narrative is to provide a realistic clinical scenario of an encounter with a patient with yaws. The objective for this scenario is part of a set of four simulations in which participants will have a greater understanding of the dermatologic aspects of neglected tropical diseases. |
| **Primary Learning Objectives** | The learning objectives for this simulation are that patients will be able to better:   - Describe, assess, and diagnose patients presenting with common neglected tropical diseases with dermatologic manifestations. - Learn the nomenclature used to describe dermatologic manifestations - Improve clinical skills and communication through interactions with standardized patients - Understand the global health significance of these tropical diseases - Work in an inter-professional group, in a way that respects patient autonomy while limiting medical jargon |
| **Critical Actions** | 1. Participants will work together as an inter-professional team to take a thorough history of a patient presenting with yaws. 2. Participants will interact professionally with the patient. 3. Participants will use their notes regarding this patient to correctly diagnose yaws in the debrief session. |
| **Learner Preparation** | No prior information needed. |

| **Initial Presentation** | | | |
| --- | --- | --- | --- |
| **Initial vital signs** | Heart Rate: 68 bpm  Respiratory Rate: 12 breaths per minute  Temperature: 100.1^0^F  Blood Pressure: 118/76 | | |
| **Overall Appearance** | Patient is well-groomed, alert and sitting upright in the patient encounter room. On the right arm, there is a yellow crusting lesion. The room is stocked with gowns, drapes, gloves, and other physical exam components. | | |
| **Actors and roles in the room at case start** | The actor can be any gender, age and ethnicity and they will have a yellow crusting lesion on their right arm as described in the overall appearance section above. Their role is to act as a patient with yaws. May be played by health professional student or professional standardized patient. | | |
| **HPI** | **HPI:** Volunteered by patient-  -The patient noticed a small papule on the right arm approximately one week ago.  -She describes that it looked like a raspberry when it first started.  -A yellow crust started forming a few days after.  **ROS:**  -Skin changes  -Slightly enlarged lymph nodes  **Social History:**  - Patient was born and raised in Ghana and moved to the United States recently. Lives with family in a small apartment.  - Exercises regularly  - Balanced Diet  - Smokes 1pack/day  - EtOH: None.  - Occupation: Unemployed  - Education: Attending school  - Not sexually active | | |
| **Past Medical/Surgical History** | **Medications** | **Allergies** | **Family History** |
| - No past medical history, surgeries or hospitalizations. - No past pregnancies - No immunizations or preventative screening. | Herbal tea from village healer | No known allergies | None; parents and younger brother all live in Ghana still |
| **Physical Examination:** | | | |
| **General** | Well-developed, well-nourished. No acute distress. | | |
| **HEENT** |  | | |
| **Neck** |  | | |
| **Lungs** |  | | |
| **Cardiovascular** |  | | |
| **Abdomen** |  | | |
| **Neurological** |  | | |
| **Skin** | Pink to white papule with yellow crust on right arm. | | |
| **GU** |  | | |
| **Psychiatric** |  | | |

| **Instructor Notes - Changes and Case Branch Points**  Due to the nature of this simulation this section is not necessary. | | |
| --- | --- | --- |
| **Intervention / Time point** | **Change in Case** | **Additional Information** |
| *6 minutes into the interview* | *Termination of the interview* |  |

**Ideal Scenario Flow**

The simulation participants have a minute to read the information posted outside of the patient encounter room that displays vitals and physical exam findings that cannot be demonstrated on the patient. The simulation participants enter the room to a find a patient in no immediate distress, but with a significant lesion upon examination. The participants take notes regarding the dermatologic presentation of the patient, history, epidemiology, and symptoms.

At the conclusion of the six minutes per room, the patient will break character and present the participants with a fact sheet containing useful information for diagnosing dermatologic neglected tropical diseases. The simulation participants are then directed to the next patient room, where the process is repeated with a different patient encounter. The participants gather a total of five fact sheets from the four patient rooms. Using the fact sheets, they will be able to identify which disease this patient had by compiling their notes as a group.

**Anticipated Management Mistakes**

Due to the quick paced nature of the encounter, we anticipated that the standardized patient might forget to present the fact sheet to the exiting group. This did not happen, but it should be stressed to the standardized patients to not forget.

A mistake encountered during the simulation was that occasionally one individual in the group would be take a dominant role, limiting other individual interaction. This would likely not be as significant in pre-established groups or groups of individuals with the same background knowledge.
