# Supplementary material for: Dermatologic Simulation of Neglected Tropical Diseases for Medical Professionals
Source: MedEdPORTAL. 2016 Dec 31;12:10525. doi: 10.15766/mep_2374-8265.10525 (PMC6440398; doi:10.15766/mep_2374-8265.10525)
Supplement: Supplementary file 1 — A. Dengue Fever Simulation Case Template.docx B. Leishmaniasis Simulation Case Template.docx C. Lepromatous Leprosy Simulation Case Template.docx D. Yaws Simulation Case Template.docx E. Dermatological Door Sheets With Vital Signs.docx F. Standardized Patient Actor Scripts.docx G. Fact Sheets.docx H. Simulation Pictures.docx I. Postsimulation Survey.pdf [file mep-12-10525-s001.zip › E. Dermatological Door Sheets With Vital Signs.docx]

**Appendix E. Dermatological Door Sheets with Vital Signs**

**Leprosy (Lepromatous)**

Patient is a 34-year-old male that presents with a chief complaint of painless lesions on his face and trunk with associated numbness.

Vitals:

Heart Rate: 66 bpm

Respiratory Rate: 10 breaths per minute

Temperature: 98.6^o^ F

Blood Pressure: 126/82

Physical exam:

Lesions have decreased sensation to touch, temperature, or pain.

Hoarseness

Destruction of nasal cartilage

Ulnar nerve weakness

Bilateral hand edema

**Leishmaniasis (localized cutaneous – LCL)**

Patient is a 26-year-old female that presents with a chief complaint of an ulcer on her right arm.

Vitals:

Heart Rate: 68 bpm

Respiratory Rate: 12 breaths per minute

Temperature: 98.6^o^ F

Blood Pressure: 122/78

Physical exam:

Hepatomegaly

**Dengue**

Patient is a 23-year-old male patient that presents with a chief complaint of a rash and fever.

Vitals:

Heart Rate: 80 bpm

Respiratory Rate: 10 breaths per minute

Temperature: 103.0 ^0^F

Blood Pressure: 118/76

Physical exam:

Weakness

Bleeding gums

Lab results:

Lymphopenia

Leukopenia

Neutropenia

Thrombocytopenia

**Yaws**

Patient is a 15-year-old female that presents with a chief complaint of a yellow crust lesion on right arm.

Vitals:

Heart Rate: 68 bpm

Respiratory Rate: 12 breaths per minute

Temperature: 100.1 ^0^F

Blood Pressure: 118/76

Physical exam:

Bone and joint lesions
