# Supplementary material for: Dermatologic Simulation of Neglected Tropical Diseases for Medical Professionals
Source: MedEdPORTAL. 2016 Dec 31;12:10525. doi: 10.15766/mep_2374-8265.10525 (PMC6440398; doi:10.15766/mep_2374-8265.10525)
Supplement: Supplementary file 1 — A. Dengue Fever Simulation Case Template.docx B. Leishmaniasis Simulation Case Template.docx C. Lepromatous Leprosy Simulation Case Template.docx D. Yaws Simulation Case Template.docx E. Dermatological Door Sheets With Vital Signs.docx F. Standardized Patient Actor Scripts.docx G. Fact Sheets.docx H. Simulation Pictures.docx I. Postsimulation Survey.pdf [file mep-12-10525-s001.zip › F. Standardized Patient Actor Scripts.docx]

**Appendix F. Standardized Patient Actor Scripts**

**Disease:** Lepromatous leprosy

**Presenting Situation:**

Patient is a 34-year-old male that presents with a chief complaint of painless lesions on his face and numbness.

**History of Present Illness:**

The patient’s symptoms began approximately 10 years ago. Started noticing skin lesions on trunk. Additionally, he has had progressive loss of sensation in patches.  Swelling in hands and slight joint pain in fingers. He is seeking help because the lesions have spread to his face. Peripheral neuropathy in hands/feet. Ulnar nerve problems (failure in movement of 4^th^/5^th^ digit). Trouble closing eyes.

**Past Medical History:**

- No past illnesses, surgeries, or hospitalizations
- No known allergies
- Medications: Advil as needed. Herbal medicines as needed.
- No immunizations or preventative screening done

**Family History:**

- Parents and two siblings are living in small village in Brazil
- No known diseases in the family

**Social History:**

- Patient was born and raised in Brazil and moved to the United States two weeks ago
- Divorced
- Exercises regularly
- Balanced Diet
- Smokes 1pack/week
- EtOH: Few beers on the weekend
- Occupation: Unemployed
- Education: Graduated from high school
- Living arrangement: Alone in small 1-bedroom apartment
- Not sexually active

**ROS:**

- Skin changes on face and trunk
- Swelling in hands and slight joint pain in fingers
- Peripheral neuropathy in hands/feet
- Ulnar nerve problems (failure in movement of 4^th^/5^th^ digit)
- Trouble closing eyes
- Hoarseness of voice
- Nose deformation

**Disease:** Yaws

**Presenting Situation:**

Patient is a 15-year-old female that presents with a chief complaint of a yellow crust on right arm.

**History of Present Illness:**

Noticed a small papule on right arm about one week ago. Looked like a raspberry when it first started. A yellow crust started forming a few days ago.

**Past Medical History:**

- No past illnesses, surgeries, or hospitalizations
- No past pregnancies
- No known drug allergies
- Medications: Herbal tea from village healer.
- No immunizations or preventative screening done

**Family History:**

- No known diseases in the family

**Social History:**

- Patient was born and raised in Ghana and moved to the United States recently
- Lives with family in a small apartment
- Exercises regularly
- Balanced Diet
- Smokes 1pack/day
- EtOH: None.
- Occupation: Unemployed
- Education: Attending school
- Not sexually active

**ROS:**

- Skin changes on right arm
- Slightly enlarged lymph nodes

**Disease:** Dengue

**Presenting Situation:**

Patient is a 23-year-old male patient that presents with a chief complaint of a rash and fever.

**History of Present Illness:**

Patient has had a rash on his back and right arm for 4 days. Patient also notices fever, joint pain, and headache. Was recently on vacation to Panama and came back last week. Is a nature enthusiast and was traveling in the rainforest.

**Past Medical History:**

- No past illnesses, surgeries, or hospitalizations
- No known allergies
- Medications: none
- No immunizations or preventative screening done

**Family History:**

- Parents and two younger brothers are living, but in small village in India
- No known diseases in the family

**Social History:**

- Exercises regularly
- Balanced Diet
- Smokes 1pack/week
- EtOH:  8 drinks/week (especially on weekends)
- Occupation: Graduate student studying botany
- Living arrangement: With roommates at a dorm
- Not sexually active

**ROS:**

- General: fever
- HEENT: Headache, pain behind eyes
- Skin changes on back and right arm
- Musculoskeletal: painful joints, muscle pain

**Disease:** Leishmaniasis

**Presenting Situation:**

Patient is a 26-year-old female that presents with a chief complaint of an ulcer on her right arm.

**History of Present Illness:**

First noticed small erythematous bite marks on arms and ankles. Thought they were just bug bites so didn’t do anything about it. Bites became larger and nodular, more white/yellow and tough skin. Then they opened up and now have pus and drainage. Was on a medical mission trip to Bolivia three weeks ago.

**Past Medical History:**

- No past illnesses, surgeries, or hospitalizations
- No past pregnancies
- No known allergies
- Medications: Advil as needed. Herbal medicines as needed
- No immunizations or preventative screening done

**Family History:**

- Mother: No health problems
- Father: Hypertension and diabetes
- One healthy younger sister (age 22)

**Social History:**

- Patient is a nursing student who is very involved in global outreach
- Exercises regularly
- Balanced diet
- Denies smoking/illicit drug use
- EtOH: Few beers on the weekend
- Occupation: Nursing student
- Living arrangement: Currently living in a home with two fellow classmates
- Not sexually active

**ROS:**

- Negative except for skin changes
