# Supplementary material for: Dermatologic Simulation of Neglected Tropical Diseases for Medical Professionals
Source: MedEdPORTAL. 2016 Dec 31;12:10525. doi: 10.15766/mep_2374-8265.10525 (PMC6440398; doi:10.15766/mep_2374-8265.10525)
Supplement: Supplementary file 1 — A. Dengue Fever Simulation Case Template.docx B. Leishmaniasis Simulation Case Template.docx C. Lepromatous Leprosy Simulation Case Template.docx D. Yaws Simulation Case Template.docx E. Dermatological Door Sheets With Vital Signs.docx F. Standardized Patient Actor Scripts.docx G. Fact Sheets.docx H. Simulation Pictures.docx I. Postsimulation Survey.pdf [file mep-12-10525-s001.zip › G. Fact Sheets.docx]

**Appendix G. Fact Sheets**

**Symptoms Fact Sheet**

**Dracunculiasis**

Systemic symptoms occur prior to the formation of a skin papule. These symptoms may include; fever, urticaria, pruritus, dizziness, nausea, vomiting, and diarrhea.^1^ The papule generally measures 2 to 7 cm, and severe pain occurs as the worm emerges.^1^ This clinical manifestation is the basis for diagnosis. Peripheral eosinophilia can occur.^1^

**Leishmaniasis**

In general, cutaneous leishmaniasis causes skin lesions, which can persist for months, sometimes years. The skin lesions usually develop within several weeks or months after the exposure but occasionally first appear years later (from trauma or immunosuppression).^2^ The lesions typically evolve from “papules to nodular plaques to ulcerative lesions, with a raised border and central depression”. ^2^ The lesions can be covered by scab or crust, or some lesions may persist as nodules.^2^ The lesions usually are painless but can be painful if they become infected with bacteria or if the lesions are near a joint.^2^ The healing process typically results in atrophic scarring.^2^

**Buruli Ulcer**

Buruli ulcer usually begins as a painless nodule less than 5 cm in diameter.^3^ Less common forms of initial skin lesions include “papules, plaques, and edematous lesions”. ^3^ The limbs are most commonly affected; other involved areas include the head, neck, trunk, and genital regions. ^3^

After days to weeks, the initial lesion breaks down and generally forms an ulcer with undermined edges. ^3^ Ulceration tends to progress slowly and painlessly, and systemic symptoms are absent unless secondary bacterial infections occur. ^3^

**Cutaneous Tuberculosis**

*Mycobacterium tuberculosis* may grow in the lungs, and can cause symptoms such as: “a bad cough that lasts 3 weeks or longer, pain in the chest, coughing up blood or sputum”.^4^ Other symptoms may include “chills, fever, night sweats, weakness or fatigue, weight loss, loss of appetite, and cutaneous rashes.”^4^ Cutaneous tuberculosis is an invasion of the skin by the same bacteria. Many types of cutaneous TB exist, and the type and severity of cutaneous TB is dependent on the immune response of the patient and the virulence of the bacteria.^5^

**Dengue Fever (DF)**

Classic dengue fever, often termed “break bone Fever” is an acute febrile illness. It is accompanied by retro-ortibal pain, headache and muscle and joint pain.^6^ The fever normally lasts for five to seven days. ^6^ Hemorrhagic manifestations occur commonly in patients with DF. Other symptoms including, petechiae rashes (macular or maculopapular), leukopenia, nausea, vomiting, diarrhea and respiratory tract symptoms including cough, sore throat and difficulty breathing”.^7^

**Yaws**

The primary lesion ("mother yaw") appears at the site of initial infection. It is usually a “localized papule that may develop into a large papilloma 2 to 5 cm in diameter or a solitary non-tender ulcer with a red, moist base reminiscent of a raspberry”.^8^ Overlying the ulceration are often yellow-brown crusts. The primary lesion is most commonly found on the “legs and ankles (65 to 85 percent of cases), but can also occur on the buttocks, arms, hands, or face”. ^8^ Healing occurs within three to six months and regresses into a scar. ^8^

As a result of lymphatic and hematogenous spread, secondary lesions may form; appearing a few weeks to two years after the primary lesion. ^8^ Arthralgia is the most common symptom of secondary yaws. Secondary skin lesions consist of “multiple smaller excrescences, sometimes resembling the primary lesion ("daughter yaws") that may ulcerate”, or as multiple “scaly patches or plaques with discoid or irregular shapes”. ^8^

**Onchocerciasis**

Onchocerciasis is classified as a subcutaneous category of diseases caused by the nematode *Onchocerca volvulus.* Freely mobile subcutaneous nodules measuring 0.5-3.0 cm are the main presenting feature, followed by ocular manifestations that develop years later.^9^ The first ocular sign of infection is the presence of microfilariae (an early form of the parasite) in the eye in the absence of any other pathology detected by slit-lamp examination. ^9^ Onchocerciasis is the second leading cause of blindness worldwide due to infection. ^9^ Additional manifestations include: sclerosing keratitis, vision loss, photophobia and eye pain.^9^

**Lepromatous Leprosy**

Those infected with a higher burden of *M. leprae* and have a weak immune response develop lepromatous leprosy. They develop numerous, painful erythematous macules, papules and or nodules lesions on all parts of the body.^10^ As the disease advances, thickening of the earlobes and hair loss occurs.^10^ If *M. leprae* invades the nasal mucosa, it can cause septal perforation or collapse (saddle nose).^10^ Patients with lepromatous disease develop generalized nerve damage.^10^ Other symptoms may include; headache, insomnia, high fever, lymphadenopathy as well as muscle and joint pain.^10^

**References:**

1. Leder K, Weller P. Miscellaneous nematodes. UpToDate Web site.<http://www.uptodate.com/contents/miscellaneous-nematodes>. Updated November 3, 2015. Accessed December 5, 2015.
2. Parasites- Leishmaniasis. Centers for Disease Control and Prevention Web site.<http://www.cdc.gov/parasites/leishmaniasis/health_professionals/>. Updated August 8, 2016. Accessed December 5, 2015.
3. Van der Werf T, Stienstra Y. Buruli Ulcer (Mycobacterium ulcerans infection). UpToDate Web site. <http://www.uptodate.com/contents/buruli-ulcer-mycobacterium-ulcerans-infection>. Updated February 18, 2016. Accessed December 5, 2015.
4. Tuberculosis (TB) Disease: Symptoms & Risk Factors. Centers for Disease Control and Prevention Web site. <http://www.cdc.gov/features/tbsymptoms/>. Updated January 7, 2016. Accessed December 5, 2015.
5. Ngan V. Cutaneous tuberculosis. DermNet New Zealand Web site. <http://www.dermnetnz.org/topics/cutaneous-tuberculosis/>. Accessed December 5, 2015.
6. Rothman A, Srikiatkhachorn A, Kalayanarooj, S. Clinical manifestations and diagnosis of dengue virus infection. UpToDate Web site. <http://www.uptodate.com.ezproxy.med.ucf.edu/contents/clinical-manifestations-and-diagnosis-of-dengue-virus-infection/contributors?utdPopup=true>. Updated April 4, 2016. Accessed December 5, 2015.
7. Symptoms and What To Do If You Think You Have Dengue. Centers for Disease Control and Prevention Web site. <http://www.cdc.gov/dengue/symptoms/>. Updated September 27, 2012. Accessed December 5, 2015.

# Mitjà O, Mabey D. Yaws, Bejel, and Pinta. UpToDate Web site. <http://www.uptodate.com/contents/yaws-bejel-and-pinta>. Updated February 4, 2015. Accessed December 5, 2015.

1. Pham H, Feldman B, Woodward M, Shah V. Onchocerciasis (African River Blindness). American Academy of Ophthalmology Web site. <http://eyewiki.aao.org/Onchocerciasis_(African_River_Blindness)>. Updated February 17, 2015. Accessed December 5, 2015.

# Scollard D, Strjewska B. Epidemiology, microbiology, clinical manifestations, and diagnosis of leprosy. UpToDate Web site.<http://www.uptodate.com/contents/epidemiology-microbiology-clinical-manifestations-and-diagnosis-of-leprosy>. Updated July 28, 2016. Accessed December 5, 2015.

**Epidemiology Fact Sheet**

**Dracunculiasis**

Dracunculiasis occurs most commonly among adults in rural settings. It has caused substantial morbidity in many parts of Africa, Yemen, and India.^1^ An intense eradication program between 1986 and 2010 reduced the number of countries with endemic dracunculiasis to four: South Sudan, Chad, Mali, and Ethiopia; Niger and Nigeria reported zero indigenous cases for the first time in 2009.^1^ A total of 126 cases were reported in 2014.^1^

**Leishmaniasis**

*Leishmania* infection is endemic in scattered foci in more than 98 countries on five continents. Globally, the annual incidence of cutaneous leishmaniasis (CL) is estimated to be 0.7 to 1.2 million new cases per year.^2^ Approximately 75 percent of CL is reported from 10 countries: Afghanistan, Algeria, Brazil, Colombia, Costa Rica, Ethiopia, Islamic Republic of Iran, North Sudan, Peru, and the Syrian Arab Republic.^2^

**Buruli Ulcer**

Buruli ulcer due to *M. ulcerans* mainly affects individuals in humid, rural, tropical regions with limited access to medical care. It has been reported in about 33 (mostly tropical) countries, with the greatest frequency in Africa, particularly in the West African countries of Côte d'Ivoire, Ghana, and Bénin (20 to 158 cases per 100,000).^3^ Cases have also been described in other locales including Mexico, South America, Papua New Guinea, and Australia. ^3^ In Victoria, Australia, and in Japan, transmission has been observed in moderate, non-tropical climates.^3^

**Cutaneous Tuberculosis**

Tuberculosis is found globally, although TB prevalence in 2015 is 42% lower than in 1990.^4^ In 2014, the African region had 28% of the world’s cases, and the South-East Asia and Western Pacific regions accounted for 58% of the new cases.^4^ India (23%), China (10%), and Indonesia (10%) had the largest number of cases. ^4^

**Dengue Fever**

Dengue hemorrhagic fever (DHF) occurs predominantly in certain parts of Asia, extending to southern China and the south of the island of Taiwan and in every South American country except Chile.^5^ The risk for exposure to *A. aegypti* is often higher in urban areas.^5^

**Yaws**

Data available from a number of endemic countries demonstrate that yaws is endemic mainly in warm, humid equatorial regions of Africa, Southeast Asia, and the Paciﬁc.^6^ Elimination of yaws has been reported in Ecuador; additional data on yaws in the Americas are limited. ^6^ West Africa is an important reservoir for yaws, particularly in Ghana (20,525 cases in 2010) and Cote d’Ivoire (3704 cases in 2010).^6^ There is also a growing number of cases in the Pacific region, including Papua New Guinea (28,989 cases in 2011), the Solomon Islands (20,635 cases in 2010), and Indonesia (5319 cases in 2011).^6^

**Onchocerciasis**

The number of infected people worldwide was estimated in 2006 to be 37 million.^7^ More than 99 percent of cases occur in 27 countries in sub-Saharan Africa.^7^ Overall, 120 million people live at risk of infection in endemic countries in Africa.^7^ Smaller foci of infection have been found in Yemen and Central and Southern America (Mexico, Guatemala, Ecuador, Colombia, Venezuela, and Brazil). ^7^ Transmission has now been eliminated or interrupted in eleven of the thirteen foci in the Americas and is ongoing in one focus in Venezuela and one in Brazil.^7^

**Lepromatous leprosy**

In the 1990s, the World Health Organization (WHO) established a goal of eliminating leprosy as a public health problem by the year 2000; "elimination" was defined as a reduction in prevalence to <1 case per 10,000 population in all endemic countries.^8^ Between 1985 and 2011, the number of registered cases fell from 5.4 million to 219,075; the prevalence rate per 10,000 fell from 21.1 to 0.37; these figures exclude Europe.^8^

The prevalence of leprosy is variable; the overwhelming majority of cases are found in developing countries. Of the 16 countries reporting more than 1000 new cases annually in 2009, the greatest numbers of new cases were seen in India, Brazil, Indonesia, Bangladesh, and Nigeria.^8^ With increasing international travel, however, patients with leprosy may present anywhere.

**References:**

1. Dracunculiasis (guinea-worm disease). World Health Organization Web site. <http://www.who.int/mediacentre/factsheets/fs359/en/>. Updated March 2016. Accessed August 5, 2016.
2. Leishmaniasis. World Health Organization Web site. <http://www.who.int/mediacentre/factsheets/fs375/en/>. Updated March 2016. Accessed August 5, 2016.
3. Buruli Ulcer (Mycobacterium ulcerans infection). World Health Organization Web site. <http://www.who.int/mediacentre/factsheets/fs199/en/>. Updated Februrary 2016. Accessed August 5, 2016.
4. Global Tuberculosis Report 2015. World Health Organization Web site. <http://www.who.int/tb/publications/global_report/gtbr2015_executive_summary.pdf?ua=1>. Accessed August 5, 2016.
5. Dengue and severe dengue. World Health Organization Web site. <http://www.who.int/mediacentre/factsheets/fs117/en/>. Updated July 2016. Accessed August 5, 2016.
6. Yaws. World Health Organization Web site. <http://www.who.int/mediacentre/factsheets/fs316/en/>. Updated June 2016. Updated June 2016. Accessed August 5, 2016.
7. Onchocerciasis. World Health Organization Web site. <http://www.who.int/mediacentre/factsheets/fs374/en/>. Updated March 2016. Accessed August 5, 2016.
8. Leprosy. World Health Organization Web site. <http://www.who.int/mediacentre/factsheets/fs101/en/>. Updated April 2016. Accessed August 5, 2016.

**Treatments Fact Sheet**

**Dracunculiasis**

There is no specific drug to treat or prevent this disease. Management involves removing the whole worm and caring for the wound.^1^ Optimal management includes the following steps: immerse the affected body part in water, apply gentle traction to slowly pull the worm, wrap the worm around a stick taking care to not break the worm, apply topical antibiotics to the wound, bandage with gauze.^1^

**Leishmaniasis**

IV Sodium stibogluconate (Pentosam) or Amphotericin B.^2^

**Buruli Ulcer**

Combination treatment of Rifampin and Streptomycin.^3^

**Cutaneous Tuberculosis**

Combination treatment of Rifampin, Isoniazid, Pyrazinamide, Ethambutol.^4^

**Dengue Fever**

There are currently no therapies to treat dengue hemorrhagic fever, however supportive care including close management of fever, bleeding, plasma leakage and shock is the best course of treatment.^5^

**Yaws**

Intramuscular Penicillin G Benzathine or single dose oral Azithromycin.^6^

**Onchocerciasis**

Combination treatment of Ivermectin (recommended treatment) or Doxycycline.^7^

**Borderline Leprosy**

Combination treatment of Dapsone, Rifampin, Clofazimine.^8^

**References:**

1. Parasites- Dracunculiasis (also known as Guinea Worm Disease). Centers for Disease Control and Prevention Web site. <http://www.cdc.gov/parasites/guineaworm/treatment.html>. Accessed December 5, 2015.
2. Parasites- Leishmaniasis. Centers for Disease Control and Prevention Web site. <http://www.cdc.gov/parasites/leishmaniasis/health_professionals/>. Accessed December 5, 2015.
3. Buruli ulcer (*Mycobacterium ulcerans* infection). World Health Organization Web site. <http://www.who.int/mediacentre/factsheets/fs199/en/>. Updated February 2016. Accessed December 5, 2015.
4. [Treatment of Tuberculosis. Centers for Disease Control and Prevention Web site. https://www.cdc.gov/mmwr/preview/mmwrhtml/rr5211a1.htm](file:///C:\Users\Med%20Student\Documents\GHC%20Docs\Derm%20Submit\Treatment%20of%20Tuberculosis.%20Centers%20for%20Disease%20Control%20and%20Prevention%20Web%20site.%20https:\www.cdc.gov\mmwr\preview\mmwrhtml\rr5211a1.htm). Accessed December 5, 2015.

1. Symptoms and What To Do If You Think You Have Dengue. Centers for Disease Control and Prevention Web site. <http://www.cdc.gov/dengue/symptoms/>. Updated September 27, 2012. Accessed December 5, 2015.
2. Yaws. World Health Organization Web site. <http://www.who.int/mediacentre/factsheets/fs316/en/>. Updated June 2016. Accessed December 5, 2015.
3. Treatment Onchocerciasis. Centers for Disease Control and Prevention Web site. <http://www.cdc.gov/parasites/onchocerciasis/treatment.html>. Updated May 21,2013. Accessed December 5, 2015.
4. Scollard D, Stryjewska B. Treatment and Prevention of Leprosy. UpToDate Web site. <http://www.uptodate.com.ezproxy.med.ucf.edu/contents/treatment-and-prevention-of-leprosy?source=search_result&search=leprosy&selectedTitle=2~77>. Updated December 7, 2015. Accessed December 5, 2015.

**Diagnostic Workup Fact Sheet**

**Dracunculiasis**

Dracunculiasis is diagnosed on a clinical basis^1^. A CBC may show peripheral eosinophilia.^1^ Skin biopsy rarely identifies the parasite.^2^

**Leishmaniasis**

Leishmaniasis is diagnosed based on clinical history and physical examination. Specimens from ulcerations can be collected by cleaning the lesion with soap and water or povidone-iodine.  The lesion should be scraped with a sterile blade and analyzed for PCR, histology and culture for definitive diagnosis.^3^ PCR should demonstrate *Leishmania* DNA. Histology would demonstrate presence of amastigote, which is a 1.5 to 4.0 micron rod-shaped kinetoplast.^3^

**Buruli Ulcer**

Buruli ulcers are easily recognized in endemic regions because of their chronicity and characteristic appearance of undermined edges.^4^ Therefore, buruli ulcers are typically diagnosed on a clinical basis, however, acid-fast staining can be performed to assess for *Mycobacteria ulcerans*. *M. ulcerans* can be grown on Lowenstein-Jensen or liquid media.^4^ PCR can also be performed for presence of DNA.^4^

**Cutaneous Tuberculosis**

Mycobacterial culture is the gold standard for diagnosis; stained smear using acid-fast stains, skin biopsy, tuberculin skin test and interferon-gamma assay analysis can all be used to diagnose cutaneous tuberculosis.^5^

**Dengue Fever**

The diagnosis for dengue virus infection is mostly clinical. To distinguish dengue from other febrile illnesses in populations in living in dengue-endemic areas, the presence of leukopenia, neutropenia, thrombocytopenia, elevated hepatic transaminases, and the presence of petechiae can help confirm the diagnosis of dengue.^6^ For a definitive diagnosis, serology testing for dengue fever is used to confirm acute infection. Viral RNA and nonstructural protein 1 (NS1) may also be used for diagnosis during the early stages of a dengue fever infection.^6^

**Yaws**

Direct diagnostic methods are limited by the fact that the *T. pallidum* treponemes cannot be cultured. The rabbit infectivity test (RIT) is the gold standard for demonstrating *T. pallidum infection*, but this is impractical for use in rural communities because of high costs and delayed test results.^7^ Treponemes can be identified in a wet preparation of material obtained from early lesions by dark-field microscopy.^7^Additionally, yaws can be definitively diagnosed by serological testing. A combination assay that combines RPR nontreponemal antibodies has a high accuracy for diagnosis.^8^ However, this makes it difficult to be diagnosed in an area with few resources. If these resources are not available, rely on epidemiology and clinical identification to make a diagnosis.

**Onchocerciasis**

The gold standard lab test that can be performed for onchocerciasis is demonstrating microfilariae from a skin-snip biopsy sample.^9^   The biopsy specimen should be incubated in saline for up to 24 hours, after time has elapsed one can view for presence of motile microfilariae.^10^ Moreover, if available a PCR from this sample from skin-snips or skin scratches is highly sensitive and specific.^8^ Direct examination of surgical specimens may be down from excised nodules.  The oncho-27 antigen test can also be used to test for onchocerca infections.^9^ A diethylcarbamazine patch test can be used but has a high rate of false positives.^9^ However, due to high costs, the use of these latter tests are restricted in resource limited settings.

**Borderline Leprosy**

According to the World Health Organization, the diagnostic criteria for leprosy requires ≥ 1 of the following for diagnosis: hypopigmented or reddish patches with definite loss of sensation, thickened or enlarged peripheral nerve with loss of sensation or muscle weakness, and/or detection of acid-fast bacilli on slit-skin smears or biopsy.^11^For those in countries with poor resources diagnosis can be made clinically. However, biopsy is preferred to guide treatment. ^11^

**References:**

1. Leder K, Weller P. Miscellaneous nematodes. UpToDate Web site. <https://www-uptodate-com.ezproxy.med.ucf.edu/contents/miscellaneous-nematodes?source=search_result&search=dracunculiasis&selectedTitle=1~8>. Updated October 27,2016. Accessed November 4, 2016.
2. Ridzon R. Cutaneous larva migrans. DynaMed Plus Web site. <http://www.dynamed.com/topics/dmp~AN~T115661/Cutaneous-larva-migrans>. Updated November 1, 2015. Accessed November 4, 2016.
3. Aronson N. Clinical manifestations and diagnosis of cutaneous leishmaniasis. UpToDate Web site.<https://www.uptodate.com/contents/clinical-manifestations-and-diagnosis-of-cutaneous-leishmaniasis?source=search_result&search=Leishmaniasis&selectedTitle=1~99#H83446552>. Updated August 26, 2016 . Accessed November 4, 2016.
4. S van der Werf T, Stienstra Y. Buruli ulcer (Mycobacterium ulcerans infection). UpToDate Web site.  <https://www-uptodate-com.ezproxy.med.ucf.edu/contents/buruli-ulcer-mycobacterium-ulcerans-infection?source=search_result&search=Buruli%20ulcer&selectedTitle=1~16>. Updated February 18, 2016 . Accessed November 4, 2016.
5. Handog E, Macarayo M. Cutaneous manifestations of tuberculosis. UpToDate Web site.<https://www.uptodate.com/contents/cutaneous-manifestations-of-tuberculosis?source=machineLearning&search=cutaneous%20tuberculosis&selectedTitle=1~150&sectionRank=4&anchor=H494576#H494576>. Updated April 14, 2016 . Accessed November 4, 2016.
6. Rothman A, Srikiatkhachorn A, Kalayanarooj S. Clinical manifestations and diagnosis of dengue virus infection. UpToDate Web site. <https://www.uptodate.com/contents/clinical-manifestations-and-diagnosis-of-dengue-virus-infection?source=machineLearning&search=dengue%20fever&selectedTitle=1~79&sectionRank=2&anchor=H20#H20>. Updated October 26, 2016 . Accessed November 4, 2016.
7. Mijta O, Smajs D, Bassat Q. Advances in the Diagnosis of Endemic Treponematoses: Yaws, Bejel, and Pinta. PLOS Neglected Tropical Disease Web site. <https://www.ncbi.nlm.nih.gov/pmc/articles/PMC3812090/>. Published October 24, 2013. Accessed November 4, 2016.
8. Mijta O, Mabey D. Yaws, bejel, and pinta.  UpToDate Web site. <https://www-uptodate-com.ezproxy.med.ucf.edu/contents/yaws-bejel-and-pinta?source=search_result&search=yaws&selectedTitle=1~17>.  Updated September 29, 2016. Accessed November 4, 2016.
9. Onchocerciasis Workup. Medscape Web site. <http://emedicine.medscape.com/article/224309-workup>.  Accessed November 4, 2016.
10. Murdoch M. Onchocerciasis. UpToDate Web site. Updated September 29, 2016 . <https://www-uptodate-com.ezproxy.med.ucf.edu/contents/onchocerciasis?source=search_result&search=Onchocerciasis&selectedTitle=1~25>. Updated August 26, 2016.  Accessed November 4, 2016.
11. Leprosy (Hansen disease). Dynamed Plus Web site. <http://www.dynamed.com/topics/dmp~AN~T116507/Leprosy-Hansen-disease>. Updated December 22, 2015. Accessed November 4, 2016.

**Photographs of Dermatologic Presentation Worksheet**

Removed due to photograph copyright content.
