# Supplementary material for: Dermatologic Simulation of Neglected Tropical Diseases for Medical Professionals
Source: MedEdPORTAL. 2016 Dec 31;12:10525. doi: 10.15766/mep_2374-8265.10525 (PMC6440398; doi:10.15766/mep_2374-8265.10525)

**Appendix H. Simulation Pictures**

Dengue Fever Petechial Rash


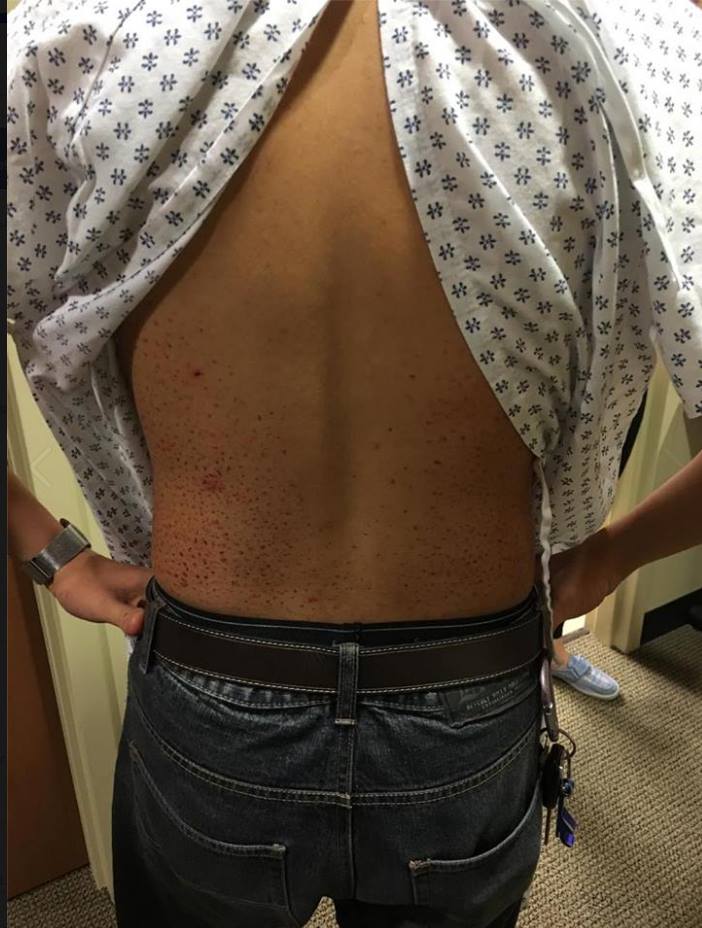


Left (Leishmaniosis), Right (Leprosy)


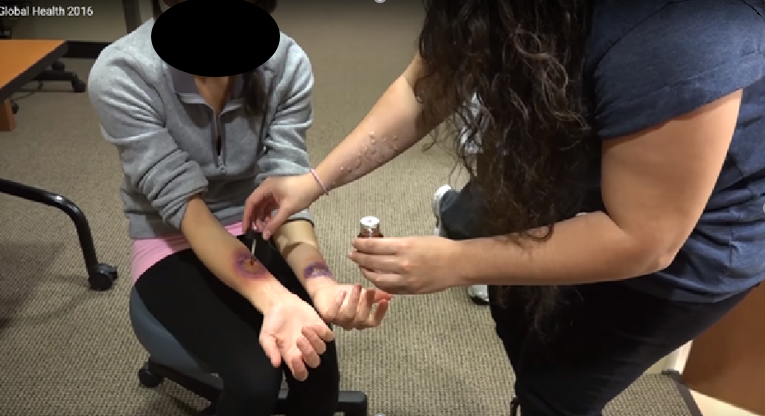

Supplement: Supplementary file 1 — A. Dengue Fever Simulation Case Template.docx B. Leishmaniasis Simulation Case Template.docx C. Lepromatous Leprosy Simulation Case Template.docx D. Yaws Simulation Case Template.docx E. Dermatological Door Sheets With Vital Signs.docx F. Standardized Patient Actor Scripts.docx G. Fact Sheets.docx H. Simulation Pictures.docx I. Postsimulation Survey.pdf [file mep-12-10525-s001.zip › H. Simulation Pictures.docx]
